# Pathogenesis of Hepatic Encephalopathy

**DOI:** 10.1155/2012/642108

**Published:** 2012-12-17

**Authors:** Irena Ciećko-Michalska, Małgorzata Szczepanek, Agnieszka Słowik, Tomasz Mach

**Affiliations:** ^1^Department of Gastroenterology, Hepatology and Infectious Diseases, Jagiellonian University Medical College, Sniadeckich Street 5, 31-531 Krakow, Poland; ^2^Department of Neurology, Jagiellonian University Medical College, Sniadeckich Street 5, 31-531 Krakow, Poland

## Abstract

Hepatic encephalopathy can be a serious complication of acute liver failure and chronic liver diseases, predominantly liver cirrhosis. Hyperammonemia plays the most important role in the pathogenesis of hepatic encephalopathy. The brain-blood barrier disturbances, changes in neurotransmission, neuroinflammation, oxidative stress, GABA-ergic or benzodiazepine pathway abnormalities, manganese neurotoxicity, brain energetic disturbances, and brain blood flow abnormalities are considered to be involved in the development of hepatic encephalopathy. The influence of small intestine bacterial overgrowth (SIBO) on the induction of minimal hepatic encephalopathy is recently emphasized. The aim of this paper is to present the current views on the pathogenesis of hepatic encephalopathy.

## 1. Introduction

Hepatic encephalopathy (HE) is a spectrum of neuropsychiatric abnormalities seen in patients with liver dysfunction after exclusion of other known brain diseases [1]. It can be the result of: acute liver failure, portosystemic bypass without hepatocellular disease or liver cirrhosis, and portal hypertension or portosystemic shunts [1]. HE manifests by the broad spectrum of neuropsychiatric disturbances such as: defects in cognitive, emotional, behavioral, psychomotor, and locomotive functions [2]. The diagnosis of overt HE is based on clinical examination. The diagnosis of minimal hepatic encephalopathy (MHE) is difficult and is based on psychometric tests [3, 4]. MHE is defined as a condition in which in patient with liver cirrhosis (regardless of etiology) the number of measurable neuropsychiatric disorders is found at a normal mental and neurologic status to clinical examination [5]. MHE was previously called early, low grade, latent, or subclinical hepatic encephalopathy [6].

## 2. Epidemiology of Hepatic Encephalopathy

Symptoms of overt HE are reported in approximately 30–45% of patients with liver cirrhosis and 10–50% of patients with transjugular intrahepatic portosystemic shunts (TIPS) [7]. Incidence of MHE is estimated by various authors at about 30–84% of patients with liver cirrhosis, depending on the applied diagnostic methods and examined population [6, 8, 9]. Romero-Gómez et al. studies have shown the presence of MHE at 53% of examined cirrhotic patients, 30% developed overt encephalopathy in the course of observation, whereas 84% of patients with overt encephalopathy had MHE in the history of disease [10]. Incidence of MHE in Polish population of patients with liver cirrhosis is estimated at 17,6–31.3% [11].

Predisposing factors for the development of HE are alcohol consumption, high levels of ammonia, zinc and branched chain amino acids, the presence of esophageal varices, and MHE [10]. Electrolyte abnormalities, bleeding into the gastrointestinal tract, infections, high protein diet, diuretics, and sedatives may stimulate the development of HE [12].

Patients with HE can present a number of clinical symptoms, which intensity increases with the progress of HE, such as: changes in personality, impaired sleep-wake cycle, attention, concentration, cognitive, and motor functions, such as psychomotor slowing, asterixis [1, 13, 14]. These symptoms can be present chronically, and exposure to factors stimulating the development of HE can lead to coma and death [15].

## 3. Pathogenesis of Hepatic Encephalopathy

According to previous studies, hyperammonemia is the main factor responsible for the brain abnormalities in HE [16, 17]. Several mechanisms that explain the influence of ammonia on the central nervous system (CNS) have been proposed such as: specific interactions between brain endothelium and astrocytes, modification of transport across the blood-brain barrier, changes in energy metabolism, a direct neurotoxic effect on astrocytes, and neuronal membranes, reducing the synthesis of free glutamate with glutamatergic neurotransmission disorder [18–20].

Ammonia is a major substrate for a number of enzymatic reactions in the brain and is also a product of some other reactions [17]. Children with congenital urea cycle enzyme defect have the high serum ammonia concentration, which—if left untreated—leads to the development of severe neurological symptoms, seizures, and coma, and at those who survived to mental retardation and paralysis of the brain [17]. A full urea cycle takes place in the liver, although some of the steps may be carried out in other tissues, including the brain [17]. It was shown that in the liver cirrhosis, the activity of urea cycle enzymes and glutamine synthetase in the brain decreases [17, 21]. As the brain is not equipped with an effective urea cycle, the ammonia is removed mainly in the process of glutamine (Gln) synthesis from glutamate (Glu) and ammonia, with the participation of glutamine synthetase localized almost exclusively in astrocytes [22]. Astrocyte swelling secondary to Gln osmotic effects consequently leads to the brain edema [23]. Hence the conception of the therapy with brain glutamine synthetase inhibitor is discussed [24].

Ammonia influences also other mechanisms leading to development of hepatic encephalopathy such as: impaired blood-brain barrier, changes in neurotransmission, proinflammatory cytokines, oxidative stress, abnormalities in GABA-ergic or benzodiazepine pathways, impaired energy metabolism of the brain, and impaired cerebral blood flow [6, 19, 25–27].

### 3.1. Ammonia and Blood-Brain Barrier

Blood-brain barrier (BBB) is formed by the endothelial cells that line cerebral microvessels [18, 28]. BBB plays an important role in the regulation of brain microenvironment homeostasis necessary for a stable and coordinated neuronal activity [29]. It protects the brain from harmful factors such as fluctuations in the blood plasma components and neurotransmitters, penetration of xenobiotics and toxins [29, 30]. BBB enables the selective transport of substances from the blood to the brain by diffusion and active transport across the endothelial cells [29, 30]. BBB plays a key role in supplying nutrients to the brain and removing unnecessary products, regulates ion homeostasis, and allows to maintain a separate pool of neurotransmitters and neuroactive substances in the CSN, in peripheral tissues and in the blood [18, 31, 32]. A number of substances such as ammonia, serotonin, bradykinin, adenosine, purine nucleotides, interleukins, free radicals, nitric oxide, and steroids may influence the brain endothelium function and tightness of BBB [18, 25].

Ammonia is a neurotoxin responsible for HE development via a direct effect on the metabolism and functions of the CNS and influencing the passage of various molecules across the blood-brain barrier, transport of branched chain amino acids, and aromatic amino acids (AA), which inflow is increased due to the formation of Gln in the process of ammonia detoxification [25].

Disturbances of AA transport affect the brain catecholamine synthesis (serotonin and dopamine) and the production of “false neurotransmitters” (octopamine and fenylethylamine), resulting in impaired GABA-ergic, serotonergic, and glutamatergic neurotransmission [15, 25].

### 3.2. Hyperammonemia and Acute Liver Failure

In the acute liver failure (ALF), which can be the result of hepatotropic viral infection or toxic injury, liver detoxification function is abruptly reduced due to the massive necrosis of hepatocytes. This leads to the hyperammonemia and development of HE, characterized by rapid progression of symptoms from discrete changes in mental status to stupor and coma.

Neuropathological studies revealed that the main cause of death in ALF was cerebral herniation with brain edema as the result of astrocyte swelling [33–36].

Pathogenesis of astrocytes swelling in the ALF is not fully understood. Hyperammonemia, brain congestion, inflammation in brain tissue, and systemic inflammatory response play an important role in astrocyte swelling [36]. At the initial stage patient has a normal or elevated intracranial pressure, which can be “controlled” using mannitol, but further progression of liver failure leads to step “uncontrolled,” requiring liver transplantation [37]. 

A number of animal studies evaluating blood-brain barrier integrity in the ALF have been carried out. The results indicate a multifactorial mechanism of encephalopathy and cerebral edema, in the pathogenesis of which the blood-brain barrier impairment is essential [38–43].

There are much less research on this subject carried out in humans.

Tofteng and Larsen using cerebral microdialysis technique studied biochemical changes in the brain of the patient with ALF during liver transplantation [44]. They showed an increase lactate concentration in the extracellular space with the proper saturation and increased glutamate and glycerol concentrations that have decreased after the transplantation. This indicate disturbances in glutamate neurotransmission and the lactate flow through the blood-brain in ALF [44].

Kato et al. examined brain biopsies of 9 patients who died of ALF using scanning electron microscopy and showed the presence of changes indicating that cytotoxic mechanism with cellular edema seems to be the main cause of the brain edema [45]. Vasogenic mechanism with impaired permeability of the BBB appears to be less important in the development of cerebral edema in ALF [45].

Kumar et al. analyzed the influence of arterial hyperammonemia on complications and outcomes in a group of 295 patients with ALF [46]. They found that persistent arterial hyperammonemia increases the risk of complications and mortality in patients with ALF [46]. Acute ammonia neurotoxicity, that can provoke seizures in patients with ALF is the result of increased release of glutamate in the neuronal synapses with excessive activation of glutamate receptors, especially the NMDA (N-methyl-D-aspartic receptor) [47].

### 3.3. Neurotransmission in Hepatic Encephalopathy

According to Albrecht et al. in HE associated with chronic liver disease, there is an imbalance between excitatory and inhibitory neurotransmission [47]. Predominance of inhibition is due to reduced expression of Glu receptors, resulting in decreased glutamatergic tone [47]. Additionally, inactivation of glutamate transporter GLT-1 in patients with hyperammonemia causes impaired Glu reuptake into astrocytes with subsequent excessive extrasynaptic accumulation of Glu [47].

*γ*-aminobutyric acid (GABA) is another factor increasing inhibitory neurotransmission through various mechanisms: increased levels of endogenous benzodiazepines, increased availability of GABA at GABA-A receptors due to enhanced synaptic release of the amino acid, direct interaction of increased level of ammonia with the GABA-A-benzodiazepine receptor complex, and ammonia-induced upregulation of astrocytic peripheral benzodiazepine receptors (PBZR) [47].

Another hypothesis assumes that the HE can be due to the inhibition of the complex GABA-benzodiazepine receptor by benzodiazepine-like ligands, that have high affinity for these receptors [48]. This theory can be confirmed by the fact that use of flumazenil-competitive antagonist of benzodiazepine receptor significantly improves the clinical status of patients [49].

### 3.4. Hyperammonemia and Neurosteroids

Hyperammonemia may also be responsible for the increase of neurosteroids concentration [15, 50]. In experimental studies adding ammonia to the culture of astrocytes, increases pregnenolone level [51, 52].

Ahboucha et al. demonstrated increased concentration of allopregnanolone strong inhibiting neurosteroide that stimulates mitochondrial peripheral-type benzodiazepine receptors (PTBR) in the brain of patients with HE [53]. The elevated neurosteroids level stimulating the GABA-A receptors may be responsible for increased GABA-ergic tone observed in HE [15, 54]. This mechanism may explain reduced motor skills, coordination problems, psychomotor slowing hypokinesia, and tremor observed in HE [55]. These motor function disorders may disturb daily functioning of patients [56].

### 3.5. Hyperammonemia and Oxidative Stress

Hyperammonemia may also have a direct toxic effect on the membranes of neurons [19]. Studies in patients with portosystemic anastomosis showed a disproportionately high level of ammonia in some regions of the brain such as cerebral cortex, which may impair the integrity of astrocytes [20].

Study of Sinke et al. on astrocyte cultures proved involvement of nuclear factor *κ*B (NF*κ*B), activated by oxidative stress, in ammonia-induced astrocyte swelling. The activation of NF*κ*B was associated with increased inducible nitric oxide synthase (iNOS) protein expression and the subsequent generation of nitric oxide (NO) [57].

According to Nörenberg et al., oxidative stress induced by ammonia is a major pathogenic factor in the ALF HE pathogenic and causes a whole cascade of events leading to cell swelling and brain edema [58]. However, encephalopathy in chronic liver disease is not accompanied by cerebral edema [58, 59]. Perhaps it is due to the fact that, together with accumulation of water in astrocytes as the result of GLN osmotic effect, the myoinositol (Ins)-organic osmolite is released from astrocytes, so its concentration in astrocytes decreases preventing change in the cell volume [60].

### 3.6. Hyperammonemia and Neuroinflammation

Neuroinflammation is a new element in the pathogenesis of HE described in animal models, which seems to play an important role in the development of cognitive impairment, that can persist after liver transplantation [61]. 

Shawcross et al. study in patients with liver cirrhosis have shown that inflammation and inflammatory mediators may significantly modulate ammonia influence on CNS (significant deterioration in psychometric test and improvement after the resolution of inflammation) [62]. The inflammation is an important factor determining the presence and severity of neuropsychological dysfunction in MHE caused by ammonia, that is, more significant in more severe inflammation [63].

A significant increase of TNF-*α* and IL-6 proinflammatory cytokines in serum of patients with MHE was noticed [26]. 

Alvarez et al. study on the astrocyte cultures indicated that proinflammatory cytokines such as TNF-*α*, IL-1*β*, IL-6, and IF-*γ* and ammonia induce increase of the mitochondria permeability and may be an important factor in the pathogenesis of HE [64]. 

Increased mitochondrial permeability transition results in reduction of ionic gradients and enhance mitochondrial dysfunction, leading to brain energetic disorders that could be a potential target for therapy [64–67].

Ammonia has a neurotoxic effect on brain astrocytes, additionally abnormalities in the cell energetic level and oxidative stress intensify HE. Change of astrocyte mitochondrial membrane permeability under the influence of ammonia and Gln may be an important mechanism for the formation of cerebral disorders associated with HE [66, 67]. Neuropathological disorders in both acute and chronic liver damage involve mainly astrocytes [68].

Astrocytes, constituing about 1/3 the of the cerebral cortex volume and playing a crucial role in the blood-brain barrier, are involved in maintaining electrolyte homeostasis, remove free radicals, and are responsible for the delivery of nutrients and neurotransmitter precursors to neurons [69]. Astrocytes play the role in the maintenance of both the ion concentration and the volume of water and thus the osmolarity of the extracellular space of the brain, and due to large capacity and ability to adjust and maintain a constant volume of the brain [68]. Hyperammonemia causes swelling of astrocytes, microglia activation, and the development of Alzheimer type II astrocytosis [70, 71]. Particularly change in astroglial morphology is characteristic for HE: edema and the presence of cells having the phenotype of Alzheimer's type II astrocytes, with a simultaneous change in the expression of genes encoding regulatory proteins supervising energy state, the cells volume, and neurotransmission [71].

Alzheimer type II astrocytes are found in the gray matter and white matter of the brain and subcortical nuclei and may have various forms suggesting hyperplasia [70]. Number of Alzheimer Type II astrocytes correlate with the encephalopathy intensity [19, 70].

Studies using the electron microscope in an animal model of portosystemic hepatic encephalopathy have shown that astrocytes before coma characterizes increased amount of cytoplasm, proliferation of mitochondria and endoplasmic reticulum, and glycogen accumulation in the cell cytoplasm, while in a coma Alzheimer's type II astrocytes was observed with the presence of degenerative changes in mitochondria and the presence of large pale nuclei with visible nucleoli [72]. These observations may suggest that ammonia initially induces astrocyte metabolic activity and subsequently the development of gliopathy. The presence of Alzheimer type II astrocytes may be responsible for the irreversibility of the changes [50]. As a result of exposure to ammonia, some profound changes occur in astrocytes, concerning the uptake of neurotransmitters and ions that change the properties of astroglial and causing its dysfunction- primary gliopathy, resulting in encephalopathy [33, 50, 70].

### 3.7. Ammonia and Brain Energy Metabolism

Another cause of HE may be dysfunction of neurons as a result of abnormal interactions between neurons and astrocytes and impairment of brain energy metabolism [65, 67]. Ammonia modifies transport of nitric oxide precursors, arginine, and ornithine (amino acids binding ammonia) across the blood brain barrier and affects the transport of energy substrate for brain, creatine, and glucose [25].

Cerebral energy metabolism and the synthesis of Gln depend on glucose supply to the brain, and the functional activity of the brain depends on the degree of glucose utilization [73]. Changes of glucose utilization in the brain were noticed in patients with liver cirrhosis: reduction in the cerebral cortex and increase in the basal ganglia and cerebellum, that could be responsible for cognitive dysfunction [74, 75].

Yazgan et al. reported significant reduction in blood flow through the thalamus and increase the flow through the frontal lobes of patients with cirrhosis compared with the healthy volunteers [76]. Another study described redistribution of blood flow from the cerebral cortex to subcortical areas in patients with liver cirrhosis [75]. Studies using magnetic resonance imaging showed an increase in cerebral blood flow and reduce the average flow time in the basal ganglia and the thalamus in patients with MHE, which is consistent with the concept of redistribution of blood from the cortex to the basal ganglia [27].

The results of research on the role of the local blood flow in the development of MHE are inconclusive.

### 3.8. Neurotoxic Effects of Manganese

There was also noticed accumulation of toxins in the brain, including manganese, which deposition in the basal ganglia might be the cause of hyperintense signals on T1-images in magnetic resonance imaging (MRI) [77–79]. In patients with liver cirrhosis who have portocaval anastomosis or TIPS hyperintense signals in the globus pallidus (88% of patients) as well as increase serum manganese concentration (67% of patients), and the extrapyramidal symptoms such as tremor, rigidity, and akinesia (89% of patients) were noticed [80]. Disturbances of manganese and other minerals homeostasis may account for the cognitive impairment associated with liver cirrhosis [81–83].

Kulisevsky et al. in brain MRI of patients with liver cirrhosis apart from increased signals in the globus pallidus found also brain atrophy, which did not correlate neither with the patients' age nor the disease duration and the number of points in the Child-Pugh score [84]. The positive correlation between the degree of brain atrophy and the level of ammonia was noticed [84]. A case of a patient with 23-year history of portocaval anastomosis (PCA) made after the second bleeding from esophageal varices was described. For 12 years, he had intense neuropsychiatric disorders and progressive degeneration of the brain in the course of 12 years of HE, with significant withdrawal after liver transplantation; therefore, these conditions should not be a contraindication for liver transplantation [85].

### 3.9. Small Intestinal Bacterial Overgrowth (SIBO) in the Development of Hepatic Encephalopathy

Recently the role of SIBO in the development of MHE has been emphasized [86, 87].

Gupta et al. study demonstrated a high prevalence of SIBO in patients with MHE (38.6%), giving evidence for the prokinetics, nonabsorbable antibiotics and probiotics therapeutic use [86].

Jun et al. found a very high prevalence of SIBO (81.3%) in patients with liver cirrhosis and ascites, which may suggest that SIBO may be a high risk factor for bacterial translocation in patients with ascites [87].

## 4. Conclusion

The pathogenesis of HE is complex and so far none of the proposed hypotheses emphasize the role of hyperammonemia and inflammation, and the role of proinflammatory cytokines, impaired neurotransmission, false neurotransmitters, oxidative stress, changes in cerebral blood flow, or brain energy metabolism does not completely explain all associated phenomena.

The results of research from last several years show the key role of blood-brain barrier in the development of HE-related disorders, but most of these are animal studies. Therefore, it is necessary to develop some modern diagnostic methods that allow the assessment of human brain metabolism.

Better understanding of the encephalopathy pathogenesis in patients with acute and chronic liver insufficiency, early diagnosis of MHE, and new therapies based on this knowledge would prevent the development of overt encephalopathy and neuropsychiatric disorders.
